# The Role of CC-Chemokines in the Regulation of Angiogenesis

**DOI:** 10.3390/ijms17111856

**Published:** 2016-11-08

**Authors:** Anisyah Ridiandries, Joanne T. M. Tan, Christina A. Bursill

**Affiliations:** 1 Heart Research Institute, 7 Eliza Street, Newtown, Sydney, NSW 2042, Australia; anisyah.ridiandries@hri.org.au (A.R.); joanne.tan@hri.org.au (J.T.M.T.); 2 Sydney Medical School, University of Sydney, Sydney, NSW 2050, Australia

**Keywords:** angiogenesis, chemokines, inflammation, ischemia

## Abstract

Angiogenesis, the formation of new blood vessels, is critical for survival and in the regenerative response to tissue injury or ischemia. However, in diseases such as cancer and atherosclerosis, inflammation can cause unregulated angiogenesis leading to excessive neovascularization, which exacerbates disease. Current anti-angiogenic therapies cause complete inhibition of both inflammatory and ischemia driven angiogenesis causing a range of side effects in patients. Specific inhibition of inflammation-driven angiogenesis would therefore be immensely valuable. Increasing evidence suggests that the CC-chemokine class promotes inflammation-driven angiogenesis, whilst there is little evidence for a role in ischemia-mediated angiogenesis. The differential regulation of angiogenesis by CC-chemokines suggests it may provide an alternate strategy to treat angiogenesis associated pathological diseases. The focus of this review is to highlight the significant role of the CC-chemokine class in inflammation, versus ischemia driven angiogenesis, and to discuss the related pathologies including atherosclerosis, cancer, and rheumatoid arthritis. We examine the pros and cons of anti-angiogenic therapies currently in clinical trials. We also reveal novel therapeutic strategies that cause broad-spectrum inhibition of the CC-chemokine class that may have future potential for the specific inhibition of inflammatory angiogenesis.

## 1. Introduction

Angiogenesis is the formation of new blood vessels from pre-existing blood vessels [1]. It is an essential process for development, growth, and repair. Under postnatal physiological conditions, angiogenesis is important for tissue neovascularization in response to ischemia (e.g., following myocardial infarction and in peripheral vascular disease) and in repair during wound healing. In contrast, uncontrolled regulation of angiogenesis that occurs with excessive or prolonged inflammation can lead to the promotion of pathological angiogenesis that is associated with the acceleration of diseases such as cancer and atherosclerosis. Increasing evidence implicates chemokines as key regulators of angiogenesis. Several members of the CC-chemokine class are implicated in a number of diseases in which inflammation-induced pathological angiogenesis plays an important role. Inflammatory angiogenesis is not, however, exclusive to the CC-chemokine class and members of the CXC and CX_3_C classes also play a role. Some CXC-chemokines, including those which bind to CXCR2 (and CXCR1 in humans), are also known to promote inflammation-driven angiogenesis. For example, CXCL5 and CXCL8 which bind to CXCR2 promote angiogenesis [2], while CXCL4 and CXCL10, which bind to CXCR3 inhibit angiogenesis in the context of inflammation [3]. Furthermore, in vitro studies have shown that the CX_3_C chemokine CX_3_CL1 (fractalkine) is a stimulator of angiogenic functions such as endothelial cell migration, proliferation and tubulogenesis [4].

Inflammation-driven diseases associated with angiogenesis are highly prevalent globally, with cardiovascular related disorders and cancer being the leading causes of mortality worldwide [5]. Current anti-angiogenic therapies suppress angiogenesis in all pathophysiological conditions, and while many are successful in suppressing disease progression, they often cause severe side effects including high blood pressure and gastrointestinal disorders [6,7]. This highlights the need for novel therapeutic targets and/or strategies that can specifically inhibit inflammation-driven angiogenesis while having no effect on important physiological ischemia-mediated angiogenic processes.

## 2. Conditional Regulation of Angiogenesis

**Figure 1:**
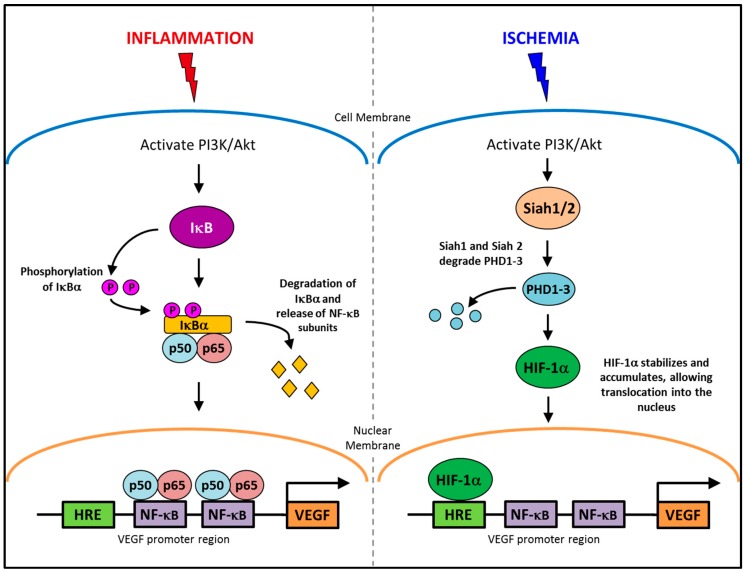
The conditional regulation of vascular endothelial growth factor (VEGF) in inflammation and ischemia. An inflammatory stimulus activates the PI3K/Akt pathway, leading to the phosphorylation of IκBα. IκBα is degraded allowing NF-κB subunits, p50 and p65 to translocate into the nucleus and activate the production of VEGF. Whereas, ischemia activates the PI3K/Akt pathway to increase the production of Siah1 and Siah2, causing the degradation of PHD1-3 preventing the targeted degradation of HIF-1α, allowing it to accumulate and then translocate to the nucleus where it upregulates VEGF production.

The regulation of angiogenesis involves a number of critical growth factors, cytokines, signaling cascades and cellular processes that are triggered in response to either an inflammatory or ischemic stimulus. Inflammatory and ischemia-driven angiogenesis are regulated via two distinctly different, yet slightly overlapping pathways [8]. Inflammation-driven pathological angiogenesis accelerates a number of disease processes, in particular tumor growth in cancer and plaque expansion in atherosclerosis through intra-plaque neovascularization [8,9,10]. Inflammatory angiogenesis also exacerbates rheumatoid arthritis [11]. A key promoter in the inflammatory angiogenic pathway is the transcription factor nuclear factor-κB (NF-κB). Whilst, in ischemia-induced physiological angiogenesis that is critical for wound healing or following a myocardial infarction to promote re-oxygenation of the heart tissues, the transcription factor, hypoxia inducible transcription factor-1α (HIF-1α), is the key promoter. Interestingly, vascular endothelial growth factor (VEGF) is a key promoter of both inflammation-driven pathological angiogenesis and ischemia-induced physiological angiogenesis. This is possible because VEGF has response elements for both NF-κB and HIF-1α in its promoter region (Figure 1), allowing for activation of its production from both signaling pathways [12,13,14,15]. HIF-1α also possesses a response element for NF-κB signaling [16]. By having both NF-κB and hypoxia response elements (HREs) in the promoter regions of HIF-1α and VEGF, it enables conditional regulation of angiogenesis. This implies for example that specific suppression of inflammatory angiogenesis will not prevent the induction of physiological ischemia-driven angiogenesis via the HREs. Whilst inflammatory and ischemia-driven angiogenesis are regulated by distinct pathways, these two forms of angiogenesis are likely to co-exist. In atherosclerosis and in tumors, as the plaque/tumor grow, cells become distant to nearby vessels creating hypoxic regions that are the initial triggers for neovascularization. Plaque/tumor neovessels then deliver inflammatory cells/cytokines to the site and inflammation becomes the predominate driving force in the progression of the disease [17].

### 2.1. Pathological Inflammatory Angiogenesis

Pathological angiogenesis is induced at sites of vascular inflammation where there has been increased recruitment of monocytes/macrophages. Macrophages are potent promoters of angiogenesis as they release pro-angiogenic factors, including VEGF, basic fibroblast growth factor (bFGF), platelet derived growth factor (PDGF), tumor necrosis factor-α (TNF-α) and interferon-γ (IFN-γ) that then stimulate excessive neovessel formation [18,19,20]. In plaques, tumors and in RA, neovessels facilitate increased transportation of more inflammatory cells, cytokines and growth factors to the site and exacerbate the disease further [9,10,21]. Neovessels in atherosclerotic plaques are particularly undesirable as they are thin walled and prone to hemorrhage making the plaque unstable [22]. Tumor neovessels also have undesirable effects and lead to metastasis as the local lymphatic systems are unable to support the tumor expansion and shunt tumor cells to neighboring lymph nodes.

An increase in inflammation can also directly cause the stimulation of angiogenesis in endothelial cells. Under inflammatory conditions, vessels form dense and highly disorganized networks [23]. These new vessels develop from existing vessels in which endothelial cells are recruited from sites adjacent to the injury [24]. These recruited endothelial cells accelerate angiogenesis either by forming part of the new vessel or by stimulating vessel growth in a paracrine fashion. At inflammatory sites in tumors, fibroblasts aid in the remodeling of the extracellular matrix (ECM) to make way for new vessels and have also been shown to have pro-angiogenic properties and promote rapid tumor growth and metastasis [21,25,26]. Due to the imbalance between the pro- and anti-angiogenic switch, pathological angiogenesis persists and exacerbates the disease, preventing its resolution.

### 2.2. Physiological Ischemia-Driven Angiogenesis

Physiological angiogenesis is primarily triggered when there is a chronic imbalance in tissue oxygen supply versus demand following injury or vessel occlusions, which restricts the supply of blood. Neovessel growth is triggered via signaling through HIF-1α. HIF-1α is regulated post-translationally by the prolyl hydroxylase domain protein family (PHD1, PHD2, PHD3). Under high oxygen conditions, PHDs are upregulated causing ubiquitination and degradation of HIF-1α [27,28]. Whilst in low oxygen conditions, E3 ubiquitin ligases Siah1 and Siah2 are upregulated resulting in the degradation of PHD1-3 leading to stabilization and accumulation of HIF-1α [29,30]. This enables the translocation of HIF-1α into the nucleus, where it complexes with the HIF-1β subunit to bind to the hypoxia response element (HRE). HIF-1α upregulates pro-angiogenic growth factors including a 30-fold increase in VEGF [29,30]. HIF-1α is essential in physiological angiogenesis. For example, inactivation of HIF-1α in mice results in abnormal vascular developmental and embryonic lethality [31,32,33,34].

Endothelial nitric oxide synthase (eNOS) is a key enzyme responsible for the promotion of angiogenesis in ischemia and is downstream of VEGF Receptor 2 (VEGFR2), the main receptor for angiogenesis, following activation by VEGF. Upon ischemic induction, eNOS stimulates the production of nitric oxide (NO), which directly promotes angiogenic processes. NO also controls the expression of growth factors such as VEGF, angiopoietins, and fibroblast growth factors and genes involved in matrix metabolism, including matrix metalloproteinases [35].

The important angiogenic functions are cell migration, proliferation and tubulogenesis, which are induced by both hypoxic and inflammatory stimuli [36,37]. Under each stimulus, endothelial cells undergo increased proliferation and migration prior to assembling into tubular structures to form new blood vessels. During angiogenesis, circulating endothelial progenitor cells (EPCs) from the bone marrow promote angiogenesis in a paracrine fashion by secreting growth factors and cytokines to activate local endothelial cells for angiogenesis [38,39,40]. EPCs have also been shown to incorporate into tubules and participate in angiogenesis directly [41,42].

## 3. Chemokines

Increasing evidence suggests that small (8–12 kDa) inflammatory cytokines known as chemokines are key regulators of angiogenesis. The main role of chemokines is to direct the recruitment and migration of cells to sites of inflammation or injury. They are divided into four classes according to the placement and number of the cysteine residues at the amino terminus. The CC chemokine group has two cysteine residues adjacent to each other, the CXC chemokine group has two cysteine residues separated by an amino acid. The CX_3_C chemokine group has three amino acids between two cysteine residues and the C chemokine group has only one cysteine residue at the amino terminus [43,44,45]. Of the four chemokine groups, the largest are the CC-chemokines consisting of 28 members in total across all species. This is followed by the CXC-chemokines (17 members), with the CX_3_C- and XC-chemokines having one and two members respectively.

Under homeostatic conditions, some chemokines such as CCL19 and CCL21, are constitutively expressed in cells to assist with basal leukocyte migration [46,47]. Other chemokines such as CCL2, CCL3, CCL4 and CCL5 are expressed when induced by an inflammatory stimulus [47]. Endothelial cells are able to generate and release chemokines under normal and inflammatory conditions and this is likely the major source of chemokines found on the luminal surface of the endothelium [48]. Once at the luminal surface, chemokines will first bind to glycosaminoglycans (GAGs) such as heparin and heparin sulfate, present on the surface of endothelial cells. Chemokines will then start to accumulate at the site of inflammation/injury via GAG attachment. This is essential, as many chemokines cannot recruit cells unless they are bound to GAGs [49]. GAGs are highly negatively charged and form an electrostatic interaction with the basic chemokine [50], tethering the chemokines to the cell surface ready for receptor interaction. GAG binding also causes oligomerization which is thought to be important in establishing chemokine activity but not receptor interaction [49,51]. Chemokine retention on the cell surface configures a concentration gradient which attracts leukocytes and monocytes to the area of inflammation or injury [52]. CC-chemokines bind to and activate inflammatory cells (e.g., monocytes, neutrophils) via binding to their cognate receptors. Receptor attachment causes a conformation change of the CC-chemokine, leading to an essential receptor and CC-chemokine N-terminal interaction that activates the inflammatory cell [44,51].

Interestingly, there is promiscuity in chemokine signaling, where chemokines in one class can bind to several receptors from the same class [53,54,55]. Conversely, receptors can have multiple ligands within a chemokine class [53,54,55]. For example in humans, CCR2 can bind CCL2, CCL7, CCL8, CCL13 and CCL16, whilst CCR5 can bind CCL3, CCL4, CCL5, CCL8, CCL11, CCL13, CCL14 and CCL16 [53,54,55]. Chemokine promiscuity is essential for the chemokine network as it allows for the robust control of the inflammatory system, such that if one chemokine is inhibited another may replace its function.

## 4. Chemokines in Angiogenesis Associated Diseases

In addition to their role in maintaining immune cell homeostasis, some chemokines have also been shown to have roles in the development of and, in particular, the promotion of angiogenesis. Several members of the CC-chemokine class including CCL1, CCL2, CCL5, CCL11, CCL15 and CCL16 [56,57,58,59,60] are involved in stimulating pathological inflammation-driven angiogenesis, with little evidence for a role in ischemia-mediated physiological angiogenesis. In contrast, a member of the CXC-chemokine class, CXCL12, uniquely plays an important role in physiological hypoxia-mediated angiogenesis.

### 4.1. CC-Chemokines in Pathological Inflammation-Driven Angiogenesis

**Figure 2:**
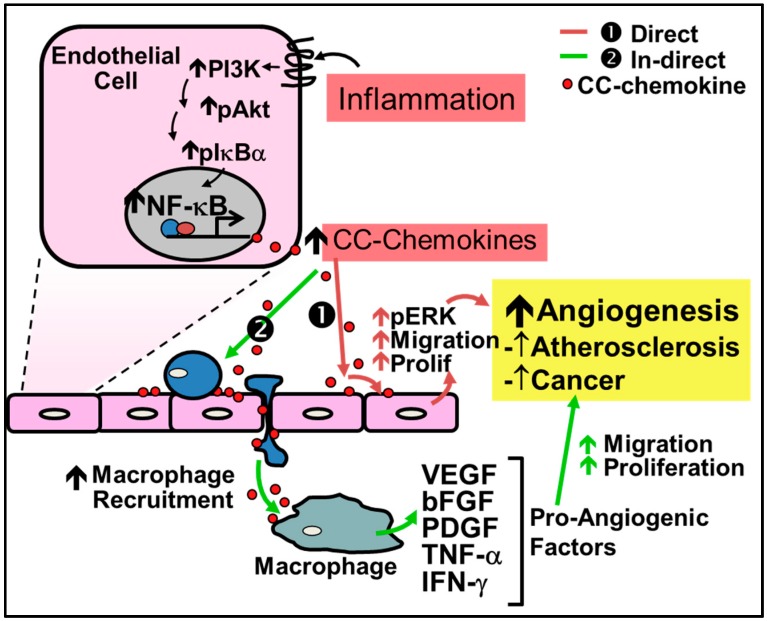
Regulation of inflammation-driven pathological angiogenesis by CC-chemokines. CC-chemokines regulate angiogenesis through two pathways: (1) the direct pathway; and (2) the indirect pathway. (1) Red Arrows - Direct stimulation by CC-chemokines promotes signaling pathways such as PI3K, MAPK, and ERK1/2 to increase nitric oxide production and promote endothelial cell proliferation and migration leading to neovessel formation; (2) Green Arrows - Indirect stimulation involves the initial recruitment of macrophages to the site of inflammation by CC-chemokines, which secrete pro-angiogenic factors such as VEGF, bFGF, PDGF, TNF-α and IFN-γ that promote angiogenesis.

CC-chemokines are increasingly implicated in disease pathologies in which inflammation-driven angiogenesis plays a key role (Figure 2). They are primarily known to promote angiogenesis indirectly by first recruiting macrophages to the site of inflammation or injury where they then release pro-inflammatory cytokines and growth factors that lead to neovessel formation. For example in liver fibrosis, angiogenesis is caused by macrophage recruitment directed by CCL2 [61]. In inflammatory conditions, CC-chemokines are secreted by a wide variety of cells including endothelial cells, smooth muscle cells and inflammatory cells.

Recent evidence demonstrates that CC-chemokines can also directly regulate inflammation-driven angiogenesis. For example, CC-chemokines have been shown to promote signaling pathways which induce inflammation-driven angiogenesis including the signaling proteins phosphatidylinositol 3-kinase (PI3K), Akt and mitogen-activated protein kinase (MAPK) and ERK1/2 to increase nitric oxide production, and endothelial cell proliferation and migration, ultimately leading to increased angiogenesis [56,62]. Stimulation of these pathways subsequently increases the production of VEGF to further augment neovascularization [56,62]. Additionally, CCL2 is associated with the increase of MMP14, essential for endothelial cell migration and neovessel formation [62]. CCL2 has also been shown to accelerate endothelialization through the recruitment of EPCs [63]. Endothelial tubule formation in vitro is increased following incubation with CCL1, CCL2, CCL11, CCL15 and CCL16 [56,57,58,59,60]. Furthermore, all CC-chemokines contain NF-κB binding motifs and their expression is dramatically increased under inflammatory conditions [54,64,65].

#### 4.1.1. CC-Chemokines in Atherosclerosis

Atherosclerosis is a chronic inflammatory disease associated with the recruitment of circulating leukocytes into the vascular wall. Inflammation-driven angiogenesis accelerates plaque formation as intra-plaque neovessels deliver more inflammatory cells to the plaque. Plaque neovessels are also fragile and prone to rupture, thereby significantly contributing to plaque instability [9]. Atherosclerosis develops over several decades and encompasses a host of cellular processes involved at various stages of disease from the initiation of plaque formation to plaque rupture, with NF-κB shown to be the key transcription factor involved throughout the entire disease progression. The initial stage of atherosclerosis involves damage to the endothelium and the activation of endothelial cells via the NF-κB signaling pathway. This results in the expression of adhesion molecules and various chemokines such as CCL2, CCL5, macrophage inflammatory factor (MIF), and CX_3_CL1 [66,67,68], that promote the recruitment of leukocytes to the vascular intima.

A number of CC-chemokines have been identified in human atherosclerotic plaques, including: CCL2, CCL3, CCL5, CCL11, CCL13, CCL18, CCL19, thereby suggesting a role for them in plaque neovessel formation [69,70,71,72]. Furthermore, targeted CC-chemokine receptor or CC-chemokine deletion studies in atherosclerosis-prone ApoE^−/−^ mice have found they develop significantly less plaque [73,74,75]. For example, Met-RANTES (an antagonist to CCL5) causes reductions in the size of atherosclerotic lesions in hypercholesterolemic LDLR^−/−^ mice [73,74,75]. Mice deficient in CCR5, the receptor for CCL5, is associated with reduced macrophage accumulation and fewer more stable plaques [76]. Conversely, overexpression of CCL2 induces macrophage infiltration and enhances atherosclerosis in ApoE^−/−^ mice [77]. Recently, a phase II clinical trial using a CCR2 neutralizing antibody MLN1202 found a decrease in C-reactive protein in patients with high risk of atherosclerosis, suggesting the potential for the suppression of inflammation, plaque angiogenesis and ultimately plaque progression [78].

#### 4.1.2. CC-Chemokines in Rheumatoid Arthritis

Studies have shown that in rheumatoid arthritis (RA), another inflammation-driven pathology, new blood vessels are abundant in the synovial tissue. Neovessels promote the infiltration of leukocytes that increase the progression of RA [11]. Previous studies have found that production of CCL2, CCL3, CCL5, and CCL20 are associated with increased leukocyte infiltration and angiogenesis in models of RA [11,79,80]. Despite the high levels of expression of CC-chemokines in RA, two separate clinical trials with MK-0812 and MLN1202, both CCR2 antagonists, were found to be ineffective [81,82]. Similarly targeting single CC-chemokine receptors CCR1 with Pfizer’s CP-481,715, and CCR5 with Maraviroc were also ineffective [83,84]. Some promising findings have, however, been demonstrated with CCX354-C, a CCR1 antagonist, that improved RA by 20% in 56% of patients after receiving a daily 200 mg dose [85].

### 4.2. Chemokines in Physiological Ischemia-Mediated Angiogenesis

**Figure 3:**
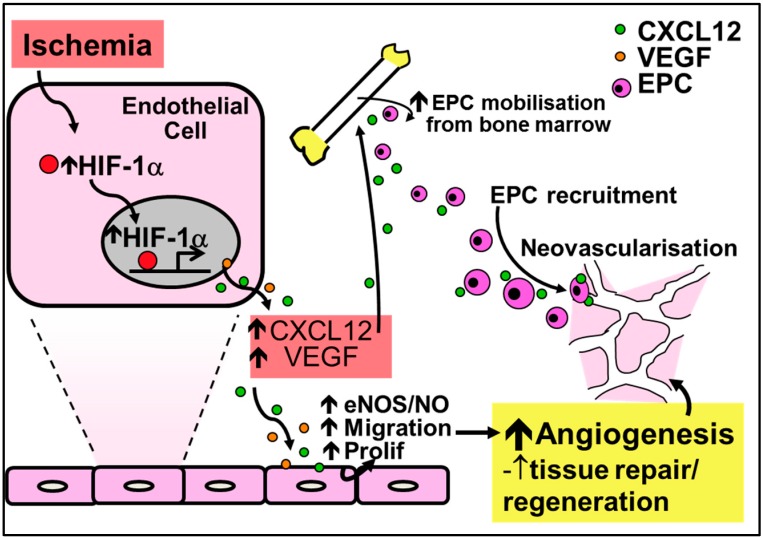
Regulation of ischemia-mediated physiological angiogenesis by the CXC-chemokine CXCL12. Increased HIF-1α in response to ischemia causes the release of CXCL12 and VEGF, leading to increased nitric oxide production and augmented endothelial migration and proliferation to promote angiogenesis. Furthermore, CXCL12 upregulates the recruitment and mobilization of EPCs to sites of ischemia. Increased nitric oxide from the induction of CXCL12 further mobilizes EPCs and increases the production of VEGF for neovessel formation.

In contrast to their critical role in inflammation-driven angiogenesis, the CC-chemokines have little involvement in ischemia-mediated angiogenesis (Figure 3). It is the CXC-chemokines, and in particular CXCL12 (also known as stromal-cell derived factor-1α, SDF-1α), that are involved in important developmental processes including hematopoiesis, organogenesis and tissue repair. This is highlighted by the fact that CXCL12 knockout mice are not viable due to the absence of vessel development [86]. HIF-1α augments the increase in expression of CXCL12 in ischemia. Consistent with this, CXCL12 expression in ischemic sites directly correlates with the degree of hypoxia [87]. Furthermore, increased CXCL12 levels have been reported in infarcted myocardium in both human and rodent studies, with increased CXCL12 levels detected as early as 1 h following ischemic induction in the myocardium or in hindlimbs, indicative of a role in the initiation of tissue repair and revascularization [88,89,90]. CXCL12 is the single natural ligand for the chemokine receptor CXCR4 [91] and the CXCL12/CXCR4 axis is important in the mobilization, migration and recruitment of EPCs to sites of ischemia [86,92,93,94,95]. Mechanistically, CXCL12 mobilizes bone marrow-derived EPCs by enhancing PI3K/Akt/eNOS activation [96]. Upregulation of eNOS further promotes EPC mobilization by increasing the production of VEGF [97].

## 5. CC-Chemokines in Cancer

Tumor angiogenesis is triggered initially by inflammation and then promoted further by hypoxia. Tumor neovessels facilitate the recruitment of inflammatory cells that express pro-angiogenic mediators. This promotes a positive angiogenic feed-forward loop, similar to that of plaque neovessels. Tumor angiogenesis is regarded as a negative prognostic variable for several malignancies including breast and prostate cancer [98,99,100]. CC-chemokines have been associated with a number of malignancies. Studies reveal that CCL2 and CCL5 expression are elevated in breast and prostate cancer [101,102]. CCL1 and CCL3 are shown to promote development of leukemia and CCL19, CCL21, and CCL25 direct tumor metastasis [103,104]. Murine cancer models have demonstrated the importance of CC-chemokines in tumor development in vivo. Inhibition of CCL2 reduced hemangioma size in nude mice inoculated with cancer cells, while infusion of Met-RANTES inhibited tumor development in murine models of breast cancer [105].

In the progression of tumor development, tumors expand to become solid tumors, causing the microenvironment to become progressively more hypoxic as cells become distant to nearby vessels. This forces cancer cells to adapt promoting tumorigenesis so oxygen and other nutrients can be delivered, expanding the tumor further. This also enhances tumor malignancy and aggressiveness, ultimately resulting in increased resistance to therapy and poor long-term prognosis. This response is induced by a series of hypoxia-mediated proteomic and genomic changes that facilitate cell survival [106]. CC-chemokines are not the only chemokines found in tumors. The CXC-chemokine class have also been shown to play a role in tumor neovascularization. For example, IL-8 (CXCL8) promotes tumor growth, angiogenesis and metastasis [107]. Furthermore, CXCL12 production is triggered in hypoxic environments in tumor cells [108] and is implicated in the promotion of tumor growth in mouse prostate tumors following overexpression of its receptor CXCR4 [109].

## 6. Anti-Angiogenic Therapies

The development of anti-angiogenic agents is of great interest due to the high prevalence of diseases associated with angiogenesis. Current anti-angiogenic agents such as VEGF inhibitors or VEGF receptor inhibitors are found to reduce atherosclerotic plaque size and tumor growth in a variety of malignancies including breast, lung and prostate cancers [10,43,110,111,112]. However, the VEGF signaling pathway is critically important in physiological ischemia-driven angiogenesis as well as other biological functions (e.g., regulation of blood pressure, activation of the coagulation cascade, and regulation of myelopoiesis [113,114,115,116]) so non-specific inhibition of VEGF in all pathophysiological contexts leads to side effects including: bleeding, vessel clots (leading to stroke or heart attack), proteinuria, severe weight loss, hypertension, diarrhea, nausea gastrointestinal perforations, and reduced wound healing [6,7,117,118]. Furthermore, the clinically available Bevacizumab (an anti-vascular endothelial growth factor, VEGF, antibody) [119] and multi-targeted tyrosine kinase inhibitors (TKIs, target the VEGF receptor), such as Sorafenib and Sunitinib [120], cause endothelial dysfunction and capillary rarefaction [6,7,117]. Both classes of drug cause clinically significant elevations in blood pressure (12%–15%), bleeding and hemorrhage—all of which are associated with impaired regenerative angiogenic responses [6,7,117]. These studies suggest that complete inhibition of angiogenesis may not be the ideal strategy to suppress pathological angiogenic diseases. This highlights the need for the development of novel therapies specifically targeting only pathological angiogenesis whilst preserving physiological angiogenesis.

Other than VEGF inhibitors, on-going clinical trials that target individual CC-chemokine receptors have shown some success, including: (1) MLN1202 that targets CCR2 and causes reductions in circulating C-reactive protein in patients with high risk of atherosclerosis; and (2) Mogamulizumab that targets CCR4 and causes reductions in regulatory T cells in patients with adult T-cell leukemia-lymphoma (ATL) and peripheral T-cell lymphoma [81,121]. However due to the promiscuity in chemokine signaling the inhibition of a single chemokine receptor may not be entirely efficacious [122,123]. An agent capable of simultaneously targeting multiple chemokines is likely to provide a more effective approach. Due to their specific role in inflammation-driven angiogenesis, targeting of the CC-chemokine class as a whole may provide a unique means of inhibiting only pathological angiogenesis without affecting critical physiological angiogenic functions.

### Broad-Spectrum CC-Chemokine Inhibition as a Strategy to Inhibit Pathological Angiogenesis

Broad-spectrum chemokine inhibitors, also called chemokine binding proteins, have been found to be expressed from a range of species including the *Schistosoma mansoni* helminth worm, the *Rhipicephalus sanguineus* tick and several viruses. Chemokine binding proteins are thought to have evolved as a way to bypass the host inflammatory response to propagate their infection. These chemokine binding proteins target multiple chemokines. For example, the *S. mansoni* chemokine binding protein (smCKBP), secreted from schistosome eggs, targets CXCL8, CCL2, CCL3, CCL5 and CX_3_CL1 [124]. Chemokine binding proteins from *R. sanguineus* tick, called Evasins, bind to (Evasin-1) CCL3, CCL4, CCL5, (Evasin-3) CXCL1, CXCL2, CXCL3, CXL5, CXCL6, CXCL8, (Evasin-4) CCL5 and CCL11 [125]. Viral protein M3 from the mouse herpes virus binds to several inflammatory chemokines across all four classes by binding to GAGs and chemokines such as XCL1, CCL2, CCL5, CXCL8 and CX_3_CL1 [126,127,128]. Vaccinia viral protein “35K” is another chemokine binding protein that uniquely binds with high affinity to the CC-chemokine class [129,130,131,132,133]. 35K has been shown to suppress inflammation-driven diseases in pre-clinical models including atherosclerosis [134,135,136], liver fibrosis and acute peritonitis [137,138]. Additionally, 35K attached to a Fc fusion protein (35K-Fc) improved pulmonary function and reduced inflammation in the lung [139]. 35K will therefore not inhibit CXCL12 which is essential for development and for tissue repair following ischemia. Broad-spectrum inhibition of the CC-chemokine class by 35K may be an alternate therapeutic strategy to specifically reduce pathological angiogenesis-associated diseases such as atherosclerosis and cancer, without the severe side effects of current non-selective angiogenic inhibitors that block angiogenesis in all pathophysiological contexts, including in ischemia.

In support of this concept, administration of CCL2 increases inflammatory angiogenesis in mice [140] and induces inflammatory angiogenesis in rabbit cornea [141]. In contrast, CCL2^−/−^ mice show no difference in capillary density compared to wild type mice in a model of myocardial ischemia [142]. Furthermore, CCR2^−/−^ mice have comparatively normal revascularization in the hindlimb ischemia model, compared to wild type mice [143]. Unpublished studies from our laboratory have found that mice injected with adenovirus overexpressing 35K (Ad35K) have reduced neovessel formation in the peri-arterial femoral cuff model of inflammation-driven angiogenesis, when compared to mice injected with control GFP adenovirus (AdGFP). In contrast, we found angiogenesis was preserved in the hindlimb model of ischemia-mediated angiogenesis following CC-chemokine class inhibition with 35K. In vitro functional assays of angiogenesis supported these findings. 35K inhibited endothelial cell proliferation, migration and tubule formation in inflammatory conditions but, conversely, had minimal effects on these angiogenic functions in hypoxia. Mechanistically, we revealed that VEGF was conditionally regulated by 35K, where VEGF is suppressed in inflammation but preserved in hypoxia (Unpublished data). The ability of 35K to inhibit inflammation-driven angiogenesis whilst preserving ischemia-mediated angiogenesis suggests it may present as an alternate therapeutic strategy for the specific inhibition of diseases associated with inflammatory driven angiogenesis.

## 7. Conclusions

In conclusion, CC-chemokines play an important role in inflammation-driven pathological angiogenesis whilst having minimal effects in ischemia-mediated physiological angiogenesis. This differential regulation of angiogenesis by CC-chemokines highlights the possibility that targeted inhibition of CC-chemokines will prevent inflammation-driven angiogenesis, whilst preserving important ischemia-mediated angiogenic processes. Current strategies targeting VEGF or single CC-chemokines/receptors have caused issues including severe side effects or ineffectiveness. This is likely due to the complete inhibition of VEGF across all pathophysiological contexts and also the promiscuity in chemokine signaling, respectively. A new approach for a more targeted strategy is broad-spectrum CC-chemokine class inhibition and there is recent evidence that it specifically inhibits pathological angiogenesis, whilst leaving the physiological angiogenesis unchanged. This may have immense benefits over current anti-angiogenic therapies.
